# Consecutive Affinity and Ion-Exchange Chromatography for AAV9 Vectors Purification

**DOI:** 10.3390/biomedicines13020361

**Published:** 2025-02-05

**Authors:** Ozgun Firat Duzenli, George Aslanidi

**Affiliations:** The Hormel Institute, University of Minnesota, 801 16th Avenue NE, Austin, MN 55912, USA

**Keywords:** adeno-associated vectors, chromatography, downstream purification, AEX

## Abstract

**Background:** Irrespective of the rapid development of AAV-based gene therapy, the production of clinical-grade vectors has a bottleneck resulting from product-related impurities such as empty and partially filled capsids, which lack a functional recombinant genome. **Methods:** In the current study, we applied the sequential affinity chromatography (AC)- and anion-exchange chromatography (AEX)-based method for purification of AAV9 vector harboring single-stranded genome encoding the fusion of firefly luciferase (fLuc)-yellow fluorescent protein (YFP) under chicken beta actin (CBA) promoter. We assessed the efficiency of two different pre-packed cross-linked sepharose and one monolithic AEX columns following AC purification to separate fully encapsulated with recombinant DNA AAV vectors from byproducts. **Results:** We showed the possibility to achieve approximately 20–80% recovery and over 90% calculated DNA-containing/empty capsid ratio depending on column and buffers composition. Additionally, we confirmed the infectivity of AAV by in vitro luciferase assay regardless of recovery method from different AEX columns. **Conclusions:** Our purification data indicate the effectiveness of dual chromatography method to obtain rAAV9 vectors with DNA-containing capsid content over 90%.

## 1. Introduction

In the past several years, adeno-associated virus (AAV) vectors rapidly became the most prevalent viral-vector-based gene delivery platform for monogenic diseases associated with blood, central nervous system, eye, and muscle function [1]. However, manufacturing challenges and safety considerations remain major obstacles to be resolved to ensure commercialization and clinical use for a wide range of diseases. Currently, seven different AAV-based gene therapy products on the market have been approved by the US FDA regulatory agency: Luxturna [2], Zolgensma [3], Elevidys [4], Hemgenix [5], Roctavian [6], Beqvez [7], and Kebilidi [8].

Human embryonic kidney (HEK293) cells and *Spodoptera frugiperda* (Sf9) cells are the most preferred production platforms, which use helper viruses such as Adenovirus (Ad), Herpesvirus or Baculovirus [9,10,11]. Regardless of the upstream platform used, rAAV production results in diverse particle distributions [12,13] and additional vector subspecies called partially filled, empty and overfilled particles [14]. In general, the problem with these subpopulations of vectors occurs due to inefficient entrapment of recombinant viral genome inside the pre-assembled AAV capsid results with production of dysfunctional AAV vectors which may correspond to ratios of 10–90% of total capsids [15]. The presence of empty and partially encapsulated capsids is not only a prominent risk for product quality, but also increases total vector load and exacerbates anti-capsid immune responses [16,17,18,19], leads to a decrease in transgene expression, and increases the risk of organ toxicity [20]. Thus, in addition to achieving the production of high AAV vector titer, reduction or elimination of empty/incomplete capsids must be considered to comply with safety requirements and increase potency of the treatments [21].

The strategies to overcome these challenges focus on either prevention of the empty capsid production during the upstream process, or removal thereof during the downstream process. The upstream process-related attempts include development of Rep hybrids/chimeras to increase the genome packaging efficiency [22,23,24], addition of small bioactive chemicals to improve viral genome titer [25], optimization of viral Rep protein expression for the production of potent and high-yield AAV particles [26], development of novel plasmid systems to reduce the number of plasmids required to increase AAV vector productivity and percentage of DNA-containing capsids [27], and bioengineering of cell lines to facilitate the control of AAV production dynamics for tuning the productivity [28]. Additionally, ensuring the integrity of ITRs is also important since it can affect the productivity, packaging efficiency, and in vivo potency [29].

The purity of the final AAV product is indispensable to prevent the occurrence of rAAV-related adverse effects such as undesired inflammatory responses [30]. In the downstream process-based approach, iodixanol- or CsCl-based gradient ultracentrifugation are the methods that exploit the differences in buoyant densities between empty and DNA-containing AAV capsids. However, these methods are hardly scalable for industrial applications [31].

The presence of DNA payload provides a lower isoelectric point (pI) to DNA-containing AAV capsids compared to empty ones [32,33]. Thus, the AEX method utilizes these differences in charge for the separation of empty and DNA-containing AAV capsids. However, the AEX method requires further optimization to improve efficiency of separation. Different approaches can be utilized including the application of linear or isocratic elution modalities [34], the use of response surface models [35], combination with cation exchange chromatography [36], using buffer compositions including novel type of salts [37,38], and application of salt and pH gradients [39,40]. In our current study, we optimized good-manufacturing-practice-compatible downstream purification process for efficient removal of empty virions from the final AAV9 vector preparation. We evaluated the efficiency of AC and AEX for removal of empty capsids from AAV9-CB-fLuc-YFP vector preparations. To this end, we first applied a capture step with AVIPure^®^-AAV9 affinity column. Subsequently, we loaded the identical fraction eluted from the affinity column onto three different anion exchange columns as follows: HiTrap^TM^ Q HP HiTrap^TM^ Capto^TM^ Q, and CIMmultus^TM^ QA. Based on our previous protocol optimization experiences, we chose a step gradient elution in AEX columns for preparative purification of AAV9 vectors.

The characteristics of AEX column materials included in this study have the following important differences. HiTrap^TM^ Q HP column contains spherical ~34 µm in size of cross-linked agarose matrix functionalized with charged groups of –CH_2_N^+^(CH_3_)_3_. Similarly, HiTrap^TM^ Capto^TM^ Q column contains spherical ~90 µm in size of highly cross-linked agarose matrix functionalized with strong charged groups of –N^+^(CH_3_)_3_. CIMmultus^TM^ QA monolithic column consists of 6 µm-pore-sized poly glycidyl methacrylate-co-ethylene dimethacrylate functionalized with strong anion exchanger quaternary amine groups. The step gradient used in our study ensures the retention of the specific biomolecules in the sample by the application of a series of mobile phases with constant and stronger composition over time, reduces running times and minimizes the volume between eluting species, while improving the resolution of closely eluting peaks [41].

In the current work, we aimed to establish an AAV9 purification platform based on sequential use of AC-AEX methods. For this purpose, we designed a purification platform consisting of AC and different alternative AEX columns with distinct properties of materials they assembled. The side-by-side evaluation of these AC-AEX pairs, which consist of different column materials and buffer compositions, showed that methods can purify AAV9 vectors with high DNA-containing capsid content and superior vector infectivity. This study offers a robust solution for the current scalability problem of the downstream process for AAV purification.

The scalability characteristic renders chromatography as an attractive method for the purification of AAV capsids. Therefore, combination of AC and AEX chromatography methods generates an adaptable purification method, which can be the production of a variety of high-quality AAV vectors suitable for preclinical and clinical testing. There are many studies evaluating different resins for AC-based purification of a variety of AAV serotypes [42,43] as a versatile and efficient alternative to the ultracentrifuge method to incorporate into AAV purification process. To support the bypassing ultracentrifuge step, AEX and size exclusion chromatography combination was also shown as a streamlined and scalable purification of AAV9 [44]. Similarly, AEX, the hydrophobic interaction chromatography, and the cation exchange chromatography following AC purification was also compared for the evaluation of purification process efficiency [36]. In addition, the effect of different resin types, buffers, additives on capsid stability during downstream processing was comprehensively investigated [45]. However, the number of studies evaluating a similar chromatography method with different columns is limited. We aimed to fill this gap by evaluating different marketed AEX columns sequentially used after the same AC column. The methods offered here will provide a general direction for using different resins and buffer compositions for AAV9 purification.

## 2. Materials and Methods

### 2.1. Cell Culture

Human embryonic kidney cells (HEK293) were maintained in complete Dulbecco’s Modified Eagle Medium (Thermo Fisher/Gibco, Manassas, VA, USA) supplemented with 10% fetal bovine serum (Thermo Fisher/Gibco, Manassas, VA, USA), 100 mM sodium pyruvate (Corning, Glandale, AR, USA) 1% penicillin and streptomycin (Genesee Scientific, El Cajon, CA, USA) at 37 °C and 5% CO_2_.

### 2.2. AAV9 Production and Purification

AAV9 vectors harboring a single-stranded expression cassette encoding firefly luciferase (fLuc) and yellow fluorescent protein (YFP) fusion driven by chicken β-actin (CBA) promoter were produced by transfection of equimolar ration of three packaging plasmids, ITRs-fLuc-YFP, Adenovirus helper, and Rep2Cap9, as previously described [46,47]. Chromatography purification was conducted on ÄKTA pure^TM^-25 chromatography system operated by UNICORN software version 7.5 (Cytiva, Piscataway, NJ, USA). First, clarified cell lysate was loaded onto AAV serotype 9 specific affinity columns (AVIPure^®^-AAV9, Repligen, Waltham, MA, USA). Subsequently, the fraction eluted from the affinity column was divided into three different fractions and each fraction applied onto three different anion exchange (AEX) columns: HiTrap^TM^ Q HP (Cytiva, Piscataway, NJ, USA, 5 mL, 17115401), HiTrap^TM^ Capto^TM^ Q (Cytiva, Piscataway, NJ, USA, 1 mL, # 29401107), CIMmultus^TM^ QA (Sartorius, Ann Arbor, MI, USA, 1 mL, #311.5113-1.3). The steps and buffer compositions in Table 1 were applied during the different column purification. The material eluted from ÄKTA was collected by using a fraction collector (Cytiva) and named as fraction (F#). Volume reduction and buffer exchange were performed using spin concentrators with 150 K molecular-weight cut-off (#AP2015010, Orbital Biosciences, Topsfield, MA, USA). Final AAV products were stored in 10 mM Tris, 100 mM sodium citrate buffer, pH 4.75. Viral genome titers were determined by qPCR with a TBGreen^®^ Advantage (#S4748, Takara Bio, San Jose, CA, USA), and primer pair specific to the CBA promoter within the AAV expression cassette: forward primer 5′-TCCCATAGTAACGCCAATAGG-3′ and reverse primer 5′-CTTGGCATATGATACACTTGATG-3′ as was described previously [47].

### 2.3. Transmission Electron Microscopy (TEM)

The method for TEM imaging was described previously [23]. Briefly, the purified–concentrated AAV vector fractions were imaged by using Tecnai G2 Spirit BioTwin electron microscope (FEI, Hillsboro, OR, USA) equipped with a 4K CCD Gatan Ultrascan camera (Gatan, Pleasanton, CA, USA) at an accelerating voltage of 120 kV and a nominal magnification of 30,000× and 49,000×. For this purpose, 4 µL of an AAV sample was placed onto 150 mesh copper grids coated with 5–6 nm Formvar/carbon EMS CF150-Cu film (Electron Microscopy Sciences, Hatfield, PA, USA) for 1 min. The grid was washed with 7 µL distilled water three times. Excess water was removed with Whatman filter paper. The sample was negatively stained with 7 µL of 0.75% uranyl acetate (SPI Supplies, West Chester, PA, USA) for 30 s. Excess staining solution was removed with Whatman filter paper, and the grid was dried at room temperature. Each AAV9 sample from AEX fractions contained higher vg titer and was captured in 6–12 electron micrographs. The average of all counting per fraction per photograph was used as the final parameter defining the ratio of DNA-containing to empty AAV capsids.

### 2.4. In Vitro Infectivity Assay

In vitro luciferase assay was performed as described previously [47]. HEK293 cells were seeded in a 96-well plate as 2.5 × 10^4^ cells/well and infected at MOI value of 20,000 vg/cell. The plate was incubated at 37 °C and 5% CO_2_ for 48 h. After incubation, wells were treated with 50 µL of in vitro luciferase substrate. Luminescence was measured by multi-well plate luminescence reader (Glomax Explorer, Promega, Madison, WI, USA) in 3 min after substrate addition.

## 3. Results

### 3.1. Affinity Chromatography-Based Capture of AAV9 Vectors

The conditions and buffer compositions used for chromatography-based purification were shown in Table 1.

Since the utilization of affinity chromatography increases the resolution and separation efficiency of the AAV purification method, we started the purification by capturing viral vectors via AVIPure^®^ affinity chromatography column which is 1 mL in volume (column volume, CV) and consists of ~50 µm in size of cross-linked spherical agarose matrix bead functionalized by synthetic peptide specific for AAV serotype 9 [48].

Initially, HEK293 cell lysate underwent three freeze–thaw cycles and Benzonase treatment. Then, cell debris was centrifuged, and the supernatant was diluted up to 100 CV with equilibration buffer (50 mM Tris, 400 mM NaCl, pH 7.5). The affinity column AVIPure^®^ was conditioned by washing 20 CV of equilibration buffer. After the sample application step, the column was washed with 20 CV of equilibration buffer to remove unbounded molecules. Since detachment of peptide protein complex takes place in low acidic conditions, AAV9 vectors bounded on the column material were eluted via elution buffer consisting of 50 mM glycine, 150 mM NaCl, pH 2. The tubes located in the fraction collector were filled with 400 µL of 1 M Tris, pH 9 to neutralize the elution with low pH to prevent AAV capsid conformational changes in low pH conditions.

**Figure 1:**
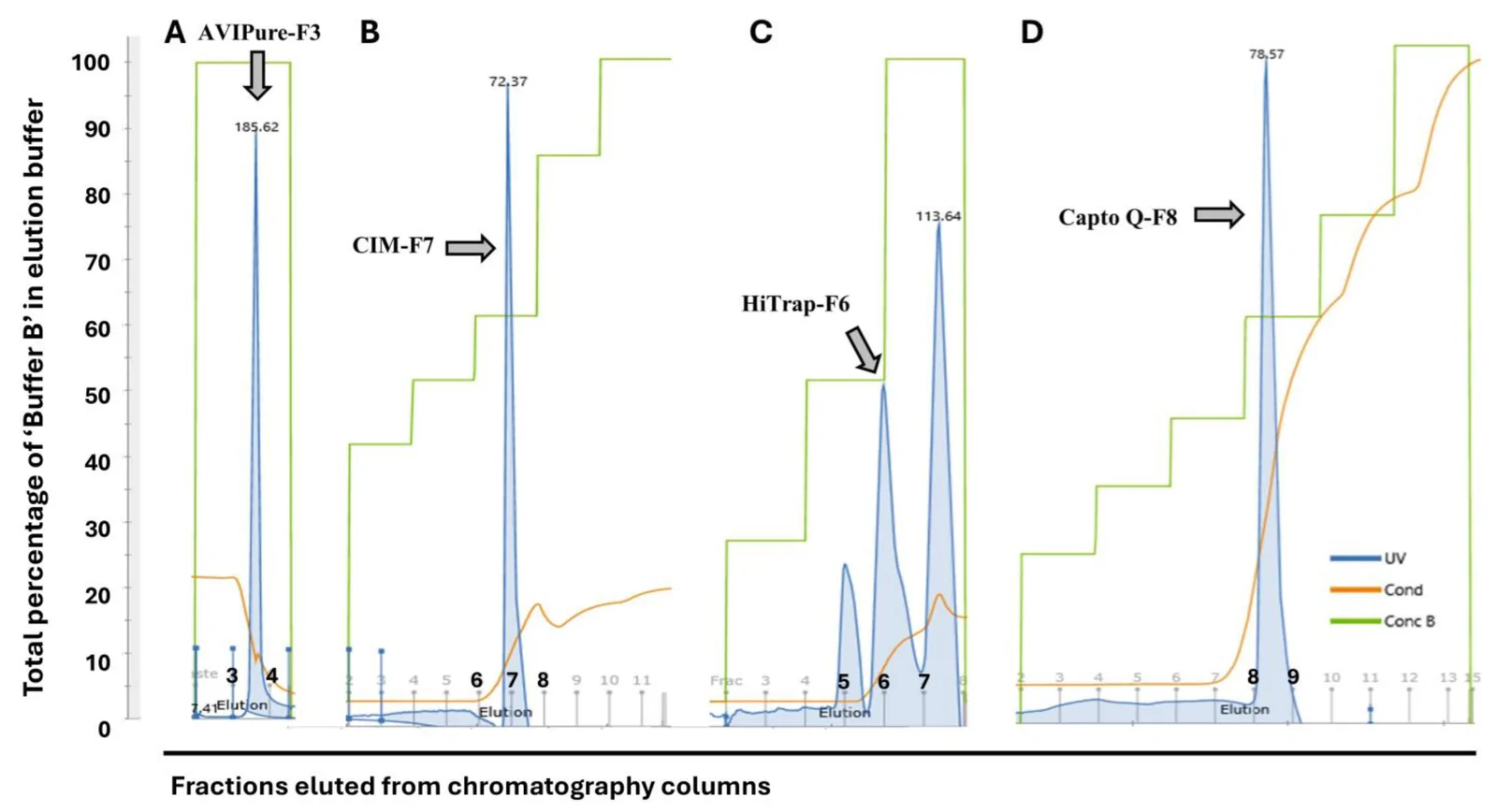
Preparative purification profiles of AAV9-CB-fLuc sample and fractions. (**A**) Affinity chromatography-based purification profile AAV9-CB-fLuc sample. pH-dependent separation with AVIPure AC column gives a single peak resolution showing that the fraction contains a protein elution. (**B**) CIMmultus monolithic AEX column-based separation of empty and DNA-containing capsid in AVIPure-F3. Salt gradient-based separation with CIMmultus monolithic AEX column gave a high single peak resolution showing that the fraction contains a protein elution. (**C**) HiTap^TM^ Q HP AEX column-based separation of AVIPure-F3 sample. The elution with step gradient gave three different peak fractions indicating the presence of proteins in the sample. (**D**) HiTap^TM^ Capto Q^TM^ AEX column-based separation of AVIPure-F3 sample. A single high peak fraction shows the protein composition. The numbers at the top of the peaks correspond to the area under the curve selected.

Chromatographic separation with AAV9 affinity column gave a single peak fraction, named AVIPure-F3 (Figure 1). We determined the viral genome quantity of AVIPure-F3 by qPCR and observed that more than 50% of total vgs were retained in AVIPure-F3. Therefore, AVIPure-F3 was divided into three parts for subsequent AEX purification.

### 3.2. Anion Exchange Chromatography Purification of AAV9 Vectors

Each of three equal parts from AVIPure-F3 was purified with a step gradient-based purification consisting of low to moderate salt compositions in three different commercially available AEX chromatography columns, which are CIMmultus^TM^ QA (Sartorius), HiTap^TM^ Q HP (Cytiva), and HiTap^TM^ Capto Q^TM^ (Cytiva).

One portion of AVIPure-F3 was loaded onto CIMmultus^TM^ QA monolithic AEX column with a column volume of 1 mL. A part of AVIPure-F3 was applied onto CIMmultus^TM^ QA monolith AEX column following the equilibration of CIMmultus^TM^ QA monolith column by passing 20 CV of equilibration buffer consisting of 10 mM BTP, pH 9, with a flow rate of 1.5 mL/min. We conducted a gradient elution consisting of 5 steps, for the separation of DNA-containing capsids from the empty capsids or capsids with partial DNA. The results presented in Figure 1B indicate that the step consisting of 40% of Buffer A (10 mM BTP, pH 9) and 60% of Buffer B (10 mM BTP, 150 mM MSO_4_, pH 9) eluted a peak of AAV attached to the column. We analyzed samples taken from the load onto column, pass-through, and fractions eluted from the column by qPCR to determine total vg in each sample. Ratios of vg titers between loaded sample and pass-through were used to calculate column-binding efficiency. The ratios of vg titers between the loaded sample and eluted fraction were used to calculate vector recovery rates within the fraction. qPCR data validated that the CIM-F7 corresponding to the single sharp peak, retained the majority of AAV vgs (Figure 1B). The calculated column binding efficiency and vector recovery rate based on CIM-F7 were 99.6% and 67.5%, respectively.

Another portion of AVIPure-F3 was loaded into HiTap^TM^ Q HP (Cytiva). The column was equilibrated with 10 mM BTP, pH 9 as 10 CV with the flow rate of 5 mL/min. Following the equilibration step, a part of AVIPure-F3 fraction was applied onto the column in 3 mL/min. After a washing step consisting of 10 CV of equilibration buffer fractions, which are 2 CV each, they were eluted from the column by using a step gradient consisting of 25, 50 and 100% of elution buffer (10 mM BTP, 100 mM MgSO_4_, pH 9), respectively, at a flow rate of 5 mL/min. The purification profile showed that the fractions that were eluted from the HiTap^TM^ Q HP were separated into three main peaks (Figure 1C). The vg titers analyzed by qPCR and the results showed that HiTrap-F6 retained the majority of AAV vgs. The calculated column binding efficiency and vector recovery rate were 99.6% and 18.7%, respectively.

The third portion of AVIPure-F3 was loaded into 5 mL HiTap^TM^ Capto Q^TM^ column. HiTap^TM^ Capto Q^TM^ column was equilibrated with 10 CV of 20 mM BTP, pH 9 with a flow rate of 5 mL/min. Subsequently, the sample was diluted with equilibration buffer, then loaded onto column with a 1 mL/min flow rate. The column was washed with 10 CV of equilibration buffer with 5 mL/min flow rate, and an elution step consisting of 25, 35, 45, 60, 75 and 100% of 20 mM BTP, 2 mM MgCl_2_, 250 mM NaOAc (pH 9) buffer was applied with 5 mL/min flow rate. An amount of 4 CV of sample was collected in each elution step. We observed a single high elution peak at Capto Q-F8 (Figure 1D). The qPCR analysis proved that the Capto Q-F8 retained most of the AAV vgs. The step gradient elution via HiTap^TM^ Capto Q^TM^ column gives 99% of binding efficiency with recovery rate in a Capto Q-F8 fraction up to 80.9%.

Overall, our results showed that optimization of purification protocol led to 99% of AAV retained in the columns during load but the recovery rate can significantly vary from approximately 20 to 80% (Table 2).

Since AAV purification involves multiple steps, some of the method-dependent problems and possible troubleshooting during the application are summarized in Table 3.

### 3.3. TEM-Based Determination of DNA-Containing Capsid Ratio Showed the Effectiveness of Different Anion Exchange Columns

**Figure 2:**
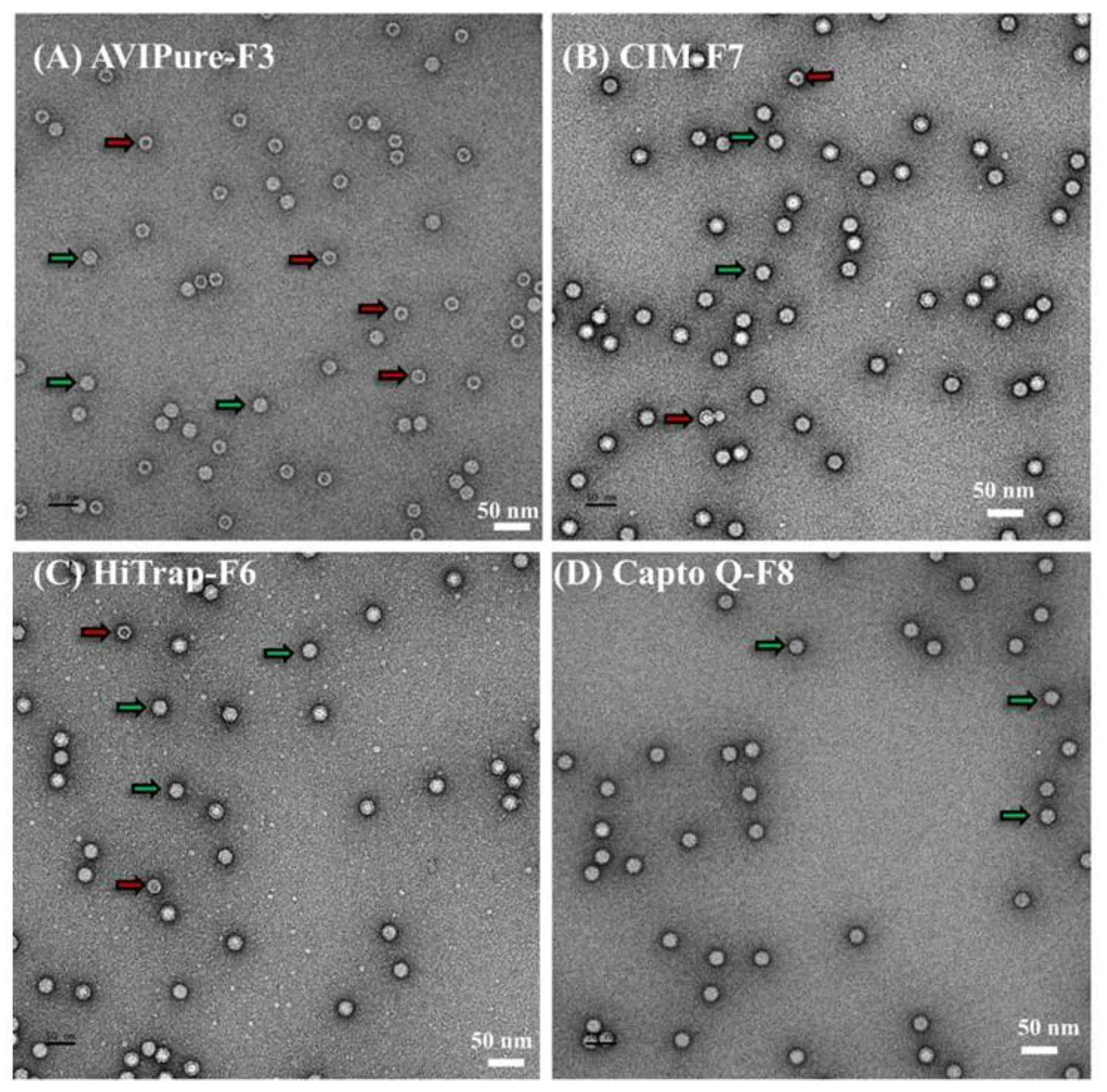
Characterization of purified AAV9-CB-fLuc fractions via TEM. Green arrows indicate DNA-encapsulated capsids (open circle) and red arrows indicate empty capsids (circle with black dot). Images represent (**A**) AVIPure-F3, (**B**) HiTrap-F6, (**C**) CIM-F7, and (**D**) Capto Q-F8 fractions.

Micro images obtained from TEM analysis showed that use of an AEX as a step of AAV9 vector purification removed the majority of empty capsids in affinity-purified AVIPure-F3 and enriched the CIM-F7, HiTrap-F6, Capto Q-F8 fractions with DNA-containing capsids (Figure 2A–D). Furthermore, according to counting AAV capsid subpopulations on TEM images, all these enriched fractions contained over 90% DNA-containing capsid: CIM-F7-98.35 ± 1.26, HiTrap-F6-96.45 ± 2.89, and Capto Q-F8 92.55 ± 7.02, respectively, which confirms the high efficiency for empty and DNA-containing capsid separation by AEX.

### 3.4. In Vitro Infectivity of AAV Does Not Depend on Purification Columns

**Figure 3:**
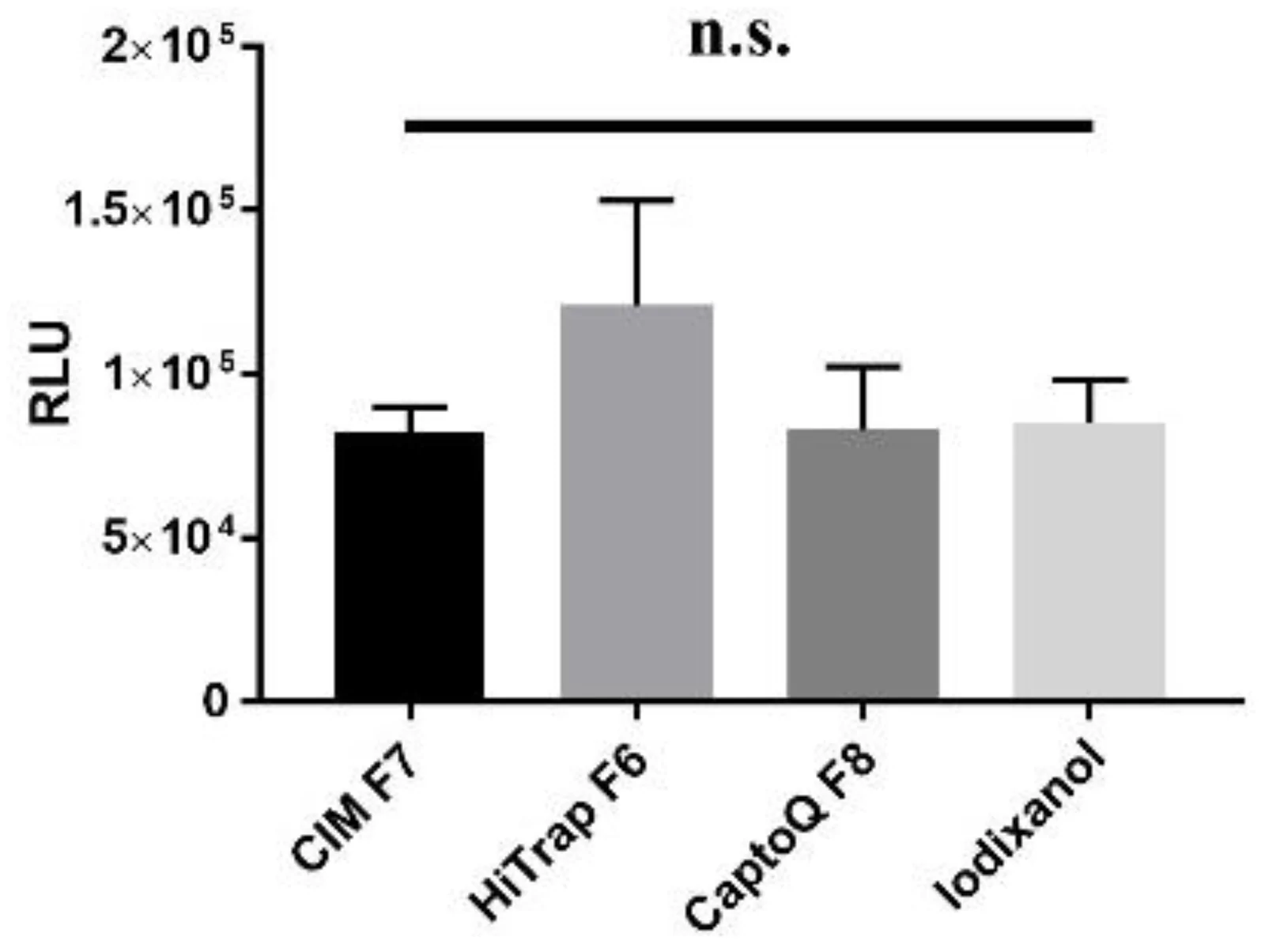
In vitro transduction assay. Samples of HiTrap-F6, CIM-F7, and Capto Q-F8 purified by AC-AEX chromatography or by iodixanol gradient ultracentrifugation. ns: non-significant, *p* > 0.05.

We tested the infectivity of all purified viruses since harsh acidic conditions or different salt concentrations used in elution steps of AC and AEX chromatography, respectively, can result in compromising AAV infectivity. The in vitro luciferase assay showed that none of the fractions eluted via tandem chromatographic method lost their infectivity compared to others or control sample consisting of AAV9-CB-fLuc-YFP vector purified by iodixanol ultracentrifugation. AAV-mediated luciferase activity normalized to vg titer was similar for all purified fractions (Figure 3). Based on the results, we concluded that neither low pH elution conditions in affinity chromatography nor electrostatic interaction between viral capsid and different AEX column material have a detrimental effect on AAV infectivity.

## 4. Discussion

The Food and Drug Administration (FDA) requires that a human gene therapy product candidate must have certain critical quality attributes (CQAs) including safety, identity, quality, purity, strength and potency. Empty capsids are considered as a typical product-related impurity, which can affect specific viral vectors activity of a drug product. The presence of empty capsids is allowed only up to a certain degree to comply with safety regulations. The purification platforms, which yield high percentage of DNA-containing capsids, will be useful for further drug release approval [49]. Additionally, systemic treatments such as Zolgensma, that consists of a recombinant AAV9 capsid harboring the cDNA of the DNA-containing-length copy of the human *SMN1* gene, require high vector doses up to 1.1 × 10^14^ vg per kg. Thus, the optimization of downstream purification process is vital for clinical AAV product development, since achieving high purity of AAV products comes with the significant loss of the viral vector [50].

The specific biomolecular interaction between the target molecule and the specific ligand, which is placed onto the stationary phase (i.e., solid matrix), gives superior selectivity attributes to affinity-based chromatographic systems [51]; additionally, characteristics such as efficient removal of non-vector based impurities, column material compatibility with vectors, and scalability are beneficial for industrial applications [52,53].

Currently, there is no AEX column that works best for all wild-type and modified AAV serotypes. Thus, purification methods for different AAV capsid compositions require the optimization of mobile phase, stationary phase, and gradient conditions. Also, separation of AAV empty and DNA-containing capsids via affinity chromatography is impossible, since empty and DNA-containing capsids have identical amino acid composition [42]. At the same time, AEX is a method allowing the separation of molecules based on their diverse charge characteristics. The mobile phase is an aqueous buffer system carrying the molecule to be separated, while the stationary phase is generally an inert organic matrix functionalized by ionizable groups including oppositely charged groups to be replaced by the molecule to be purified [54].

We showed that sequential AC-AEX chromatography method is effective to obtain AAV preparations with high DNA-containing-to-empty-capsid ratio. AEX columns, which are constructed with different materials or physical structure, can be utilized after a capture step based on their binding capacity, recovery rate, and resolution characteristics. The percentage of AAV DNA-containing capsids can vary depending on the column structure, salt composition and concentration. Overall, our results showed that the optimization of AEX purification protocol led to 80–99% enrichment of DNA-containing AAV capsids. However, HiTap^TM^ Capto Q^TM^ column gives a high percentage of recovery rate (80.9%), and CIMmultus^TM^ QA ensures a moderate to high percentage of recovery (67.5%), while HiTrap^TM^ Q HP, even though it showed the high binding capacity, had a recovery rate as low as 18.7% for the selected fraction.

Particularly, methacrylate-supported monoliths (convective interaction media, CIM) are known for their improved virus purification and concentration characteristics [52]. Here, we obtained 99% recovery of the loaded rAAV9 vectors with 10 mM BTP, 150 mM MgSO_4_ (pH 9) with CIMmultus AEX column. Consistently, Lock et al. reported [55] over 99% of the recovery rate of the loaded AAV8 with 50–150 mM NaCl gradient elution. Importantly, all the methods developed in this study can produce AAV vector preparations with high DNA-containing capsid percentage and without loss of vector infectivity. In our study, we used BTP pH 9 buffers in different AEX columns. We were not able to compare the effect of pH on the vector purification profile in different column materials. However, additional optimization of pH might not critically affect purity of AAV as Aebischer et al. [56] showed that various types of column material (monolith or nonporous), buffers and pH used in mobile phase plays a minor role in chromatographic resolution.

Additional efforts to optimize AEX might include testing of novel salt compounds for enhanced capability for separating empty and DNA-containing capsids [37], application of step gradient approaches with different buffers for the precise and accurate baseline separation by taking advantage of the retention behavior of viral capsids [56], the utilization of different salts in the elution buffer while implementing discontinuous salt gradient comprising isocratic holding steps [57], enhancement of the capsid stability under chromatographic conditions by adding divalent cations, and improvement of empty and DNA-containing capsid separation with anti-chaotropic agents [32].

Purification methods are generally dependent on the unique properties of AAV vector capsid to be purified. Even though chromatographic methods are proven to be scalable for AAV purification, they should be optimized specifically for each capsid of interest. Additionally, the elution of DNA-containing capsids together with the partial DNA-containing or empty capsids constitutes a major drawback. Affinity columns are not capable of separating empty and DNA-containing capsids, due to having an identical amino acid composition [42]. In addition, any mutation within the capsid could interrupt the interaction between the vector and affinity ligand. AEX chromatography relies on the differences in physicochemical properties between DNA-containing and empty capsids. One of these properties is the isoelectric point of AAV capsids, which differs between DNA-containing and empty capsids, but the magnitude of this difference also depends on AAV serotype [36,58], meaning that the buffer composition should be well-optimized for each AAV serotype of interest.

## 5. Conclusions

In the current work, we showed the efficacy of a tandem chromatography-based platform for rAAV9 purification. Even though we applied this system for the purification of lab-scale AAV preparations, the platform is available for any scale-up purposes, because of the nature of chromatography technology and availability of large volume columns. In addition, using different buffer compositions, pH gradients or combination of the platform with other chromatography methods might help to further increase the efficiency of separation of DNA-containing and empty capsids. Additionally, the use of affinity chromatography columns specific for other serotype(s) will help to expand the platform for the purification of different AAV vector-based drugs.

Our study emphasizes that the high vector recovery rate and excellent separation of empty and DNA-containing capsids are important characteristics of the purification platform and are parameters to consider when establishing AAV purification protocol.

## Figures and Tables

**Table 1:** The steps, volumes and buffer compositions used in affinity and anion exchange chromatography columns.

Column	Procedure	Column Volume, x	Flow Rate (mL/min)	Buffer Composition
AVIPure^®^-AAV9	1. Equilibration	20	2	50 mM Tris, 400 mM NaCl, pH 7.5
	2. Sample Application	100	1.5	HEK293 lysate with AAV9 in 50 mM Tris, 400 mM NaCl, pH 7.5
	3. Column Wash	20	2	50 mM Tris, 400 mM NaCl, pH 7.5
	4. Elution	10	2	50 mM Glycine, 150 mM NaCl, pH 2
	5. CIP	10 10 20	2 2(30 min hold) 2	a. 50 mM Glycine, 150 mM NaCl, pH 1 b. 0.5 M NaOH, c. 50 mM Tris, 400 mM NaCl, pH 7.5
CIMmultus^TM^ QA	Equilibration	20	1.5	10 mM Bis-Tris Propane (BTP), pH 9
	Sample Application	10	1.5	Post-affinity AAV9 eluate diluted into 10 mM BTP, pH 9
	Column Wash	30	1.5	10 mM Bis-Tris Propane (BTP), pH 9
	Elution (Step Gradients)	5	1.5	A: 10 mM BTP pH 9+ B:10 mM BTP, 150 mM MgSO_4_ pH 9 Step 1 = 40% B + 60% A, Step 2 = 50% B + 50% A, Step 3 = 60% B + 40% A, Step 4 = 85% B + 15% A, Step 5 = 100% B
	CIP	20	1.5	a. 10 mM BTP, pH 9
		10	10	b. 1 M NaCl
		20	1.5	c. 10 mM BTP, pH 9
Cytiva HiTap^TM^ Q HP column	Equilibration	10	5	10 mM BTP, pH 9
	Sample Application	50	3	Post-affinity AAV9 eluate diluted into 10 mM BTP, pH 9
	Column Wash	10	5	10 mM BTP, pH 9
	Elution	2	5	A: 10 mM BTP, pH 9+ B: 10 mM BTP, 100 mM MgSO_4_, pH 9 Step 1 = 25% B + 75% A, Step 2 = 50% B + 50% A, Step 3 = 100% B
	CIP	5	5	a. 2 M NaCl
		10	5	b. 10 mM BTP, pH 9
Cytiva HiTap^TM^ CaptoQ^TM^	Equilibration	10	5	20 mM BTP (pH 9)
	Sample Application	50	1	Post-affinity AAV9 eluate diluted into 10 mM BTP, pH 9
	Column Wash	10	5	20 mM BTP (pH 9)
	Elution	4	5	A: 20 mM BTP, pH 9+ B: 20 mM BTP, 2 mM MgCl_2_, 250 mM NaOAc (pH 9) Step 1 = 25% B + 75% A, Step 2 = 35% B + 65% A, Step 3 = 45% B + 55% A, Step 4 = 60% B + 40% A, Step 5 = 75% B + 25% A, Step 6 = 100% B
	Strip	5	5	2 M NaCl
	CIP	5	5	a. 2 M NaCl
		10	5	b. 1 M NaOH
		5	5	c. 2 M NaCl
		10	5	d. 20 mM BTP, pH 9

**Table 2:** Column binding efficiency and recovery rates per fraction.

Sample	Column Binding Efficiency	Vector Recovery Within the Fraction
CiMmultus F7	99.60%	67.50%
HiTrap F6	99.60%	18.70%
CaptoQ F8	99%	80.90%

**Table 3:** The possible problems can be observed during experimental procedure and recommended troubleshooting.

Problem	Solution
Flow rate fluctuates during sample application.	After cell harvesting and disruption, the content of the sample includes not only AAV vectors but also cellular components, such as membrane lipids, proteins, genomic biomolecules, etc. Centrifugation steps for the removal of cellular debris is not sufficient for clarification. Therefore, filtering samples with different mesh sizes, or application of specific columns allowing the removal of such impurities by using flow-through mode is highly recommended. Additionally, uncut gDNA can promote column plugging, since the DNA double helix has a large surface area and high viscosity, which can lead to the accumulation of the sample with high viscosity inside the column. Ensure sufficient time and concentration of Benzonase treatment.
AAV capsid does not bind to the affinity resin.	Any modification within the AAV capsid creates new AAV variants which can show less or no binding efficacy to the affinity column material. Ensure that the binding site of the antibody in the resin has not an affinity to region mutated/modified within AAV capsid.
Loss of AAV infectivity after affinity column purification.	The elution from affinity columns generally requires low pH conditions. Since the acidic environment can trigger the spatial conformational change in AAV capsid, which may lead to the loss of infectivity. To prevent the loss of infectivity, pH of environment should be adjusted to a pH value which is tolerable for the AAV vector. You can add a buffer to ensure that the eluted fraction will be maintained at an optimal pH for stability.
The co-purification of DNA-containing and empty capsids.	To increase the efficiency of the separation in AEX column, the composition of the buffers, the purification mode (linear or step gradient), and incubation time should be optimized depending on AAV capsid serotype. Additionally, the column resins or materials, regardless of the column type (affinity or ion exchange), have a lifetime so that the degradation of resin material can result in the loss of resolution, binding, and recovery capacity.

## Data Availability

The original contributions presented in this study are included in the article. Further inquiries can be directed to the corresponding author.
